# Cutaneous vasculitis and vasculopathy in the era of COVID-19 pandemic

**DOI:** 10.3389/fmed.2022.996288

**Published:** 2022-08-23

**Authors:** Carlo Alberto Maronese, Enrico Zelin, Gianluca Avallone, Chiara Moltrasio, Maurizio Romagnuolo, Simone Ribero, Pietro Quaglino, Angelo Valerio Marzano

**Affiliations:** ^1^Dermatology Unit, Fondazione IRCCS Ca' Granda Ospedale Maggiore Policlinico, Milan, Italy; ^2^Department of Pathophysiology and Transplantation, Università degli Studi di Milano, Milan, Italy; ^3^Dermatology Clinic, Maggiore Hospital, University of Trieste, Trieste, Italy; ^4^Dermatology Clinic, Department of Medical Sciences, University of Turin, Turin, Italy; ^5^Department of Medical Surgical and Health Sciences, University of Trieste, Trieste, Italy

**Keywords:** vasculitis, vasculopathy, COVID-19, COVID vaccines, cutaneous manifestation

## Abstract

Cutaneous vasculitides encompass a heterogeneous group of clinicopathological entities, which may occur as single-organ vasculitis of the skin or present as skin-limited variant of systemic vasculitis (i.e., skin-limited ANCA-associated vasculitis), and are triggered by various factors, including infections, drugs and vaccines. The COVID-19 pandemic has challenged us with a variety of both disease- and vaccine-associated skin manifestations, including vasculitis. Among the latter, cutaneous small-vessel vasculitis, previously known as leukocytoclastic vasculitis, seems to be the most reported in either scenario, i.e., natural infection and vaccination. Vasculopathy without true vasculitic changes on histology develops in but a minority of cases, mostly severe/critical COVID-19 patients, and appears to be the result of endothelial injury due to pauci-immune thromboembolic mechanisms. Herein, we provide an overview of the available literature on COVID-19-associated and anti-SARS-CoV-2-vaccine-associated cutaneous vasculitis. Although evidence is mostly limited to isolated reports, with a proportion of cases lacking histopathological confirmation, ample overlap with pre-pandemic forms is shown.

## Introduction

Cutaneous vasculitides are a heterogeneous group of inflammatory disorders affecting skin blood vessels (1). In 2018 the dermatologic addendum to the 2012 revised International Chapel Hill Consensus Conference Nomenclature of Vasculitides (D-CHCC) provided a renewed framework and nomenclature for cutaneous vasculitides (CV), devising a classification whereby cutaneous features of systemic vasculitides are discussed and cutaneous single-organ vasculitides that have no systemic counterparts are introduced (2).

Although the D-CHCC substantially furthered our understanding of CV, a wealth of new evidence has become available in the last 5 years. Provisional entities, such as macular lymphocytic arteritis also known as lymphocytic thrombophilic arteritis (LTA) (3) and recurrent cutaneous necrotizing eosinophilic vasculitis (RCNEV) (4), have been characterized more accurately. For instance, LTA has started being recognized as distinct from cutaneous polyarteritis nodosa (cPAN), presenting with a non-infiltrated, asymptomatic, and more widespread pattern of livedo racemosa (3). RCNEV, on the other hand, typically affects middle-aged Asian females, manifesting erythematous to purpuric papuloplaques, angio-oedema on the extremities and peripheral eosinophilia (4). Another provisional entity introduced in the D-CHCC is the so-called immunoglobulin (Ig) M/IgG vasculitis, a form of leukocytoclastic vasculitis involving dermal small-vessels, particularly post-capillary venules. This definition is meant for those cases of skin-limited vasculitis showing IgM/IgG deposits that are not related to cryoglobulinemia, monoclonal gammopathy and connective tissue diseases (2, 5).

The ongoing pandemic has added to the complexity of this scenario, challenging us with a variety of skin manifestations, including cutaneous vasculitis and vasculopathy, either as a direct result of the Coronavirus Disease 2019 (COVID-19) or following vaccination.

Pathogenic mechanisms are not fully understood, although the roles of a hyperactive immune response, complement activation and microvascular injury have been hypothesized.

Herein, we provide an overview of the available evidence on COVID-19-associated and anti-SARS-CoV-2-vaccine-associated CV.

## COVID-19-associated cutaneous vasculitis/vasculopathy

COVID-19-associated cutaneous manifestations include six main clinical phenotypes: (i) urticarial, (ii) maculopapular, (iii) papulovesicular, (iv) chilblain-like (6), (v) livedo reticularis/racemosa-like and (vi) purpuric vasculitic-like (7). Latency varies (7–9) and their incidence ranges between 1.8 and 20.4% of COVID-19-patients (9)—though these estimates mainly reflect data from the beginning of the pandemic.

In an Italian multicenter study investigating the clinical spectrum of COVID-19 associated cutaneous manifestations, only 13/200 adult patients presented a purpuric vasculitic pattern, with the latter being a significative risk factor for dyspnea (10)—although no clear relationship was shown with severity. Presentation may vary with livedoid features, retiform purpura and/or acro-ischemic phenomena (Table 1) (10). Systemic corticosteroids (CS) have shown some benefit, but a clear treatment protocol is lacking due to the rarity and incomplete characterization of these forms, as well as their presentation in critically ill patients (11).

**Table 1:** Clinical and histopathological features of the main cutaneous vasculitides associated with COVID-19 and/or anti-SARS-CoV-2 vaccination.

	**Clinical features**	**Histopathological features**
Cutaneous small-vessel vasculitis^*^	Palpable purpura, petechiae and/or hemorrhagic macules or (rarely) blisters. Occasionally, ulcerations can be observed. Lower extremities are commonly affected. Extracutaneous involvement is uncommon and usually mild.	Postcapillary venules are primarily affected, with endothelial swelling, a neutrophilic infiltrate with leukocytoclasia, red blood cell extravasation, and fibrinoid necrosis of blood vessel walls. Variable numbers of mononuclear cells and eosinophils may be detected. Intravascular thrombi and ischemic necrosis of the overlying epidermis may sometimes be observed. Evidence on direct immunofluorescence findings in both COVID-19- and vaccine-associated cases is inconclusive.
Skin-limited IgA vasculitis	Erythematous macules or papules evolving into palpable purpura predominantly on the lower limbs, thighs, and buttocks. Hemorrhagic bullae and targetoid lesions can also be observed.	A picture of leukocytoclastic vasculitis of small dermal blood vessels is usually seen (see above). Direct immunofluorescence demonstrates IgA deposition in vessel walls. Fibrinogen and C3 are usually present as well.
Urticarial vasculitis^**^	Erythematous, oedematous wheal-like lesions persisting more than 24 h, associated with non-blanchable purpura and resolving with hyperpigmented *sequelae*, most commonly on the trunk and proximal extremities. Burning, rather than itching is typically reported.	A picture of leukocytoclastic vasculitis of small dermal blood vessels is usually seen (see above). Lymphocytic perivascular cuffing without leukocytoclasia has also been reported in a proportion of patients.
Lymphocytic vasculitis	Maculo-papular erythemato-violaceous lesions with purpuric aspects located on lower and upper limbs. Chilblain-like appearance (i.e., “COVID toes”).	Lymphocytic perivascular cuffing of superficial and deep dermal small vessels, along with endothelial cell swelling. Dermal microthrombi may also be seen.
Pauci-immune thromboembolic vasculopathy^***^	Necrotic lesions, retiform purpura, finger or toe cyanosis, gangrene, blisters or livedoid rash.	Epidermal necrosis. Thrombotic vasculopathy of small and medium vessels in superficial and deep dermis, with sweat gland necrosis, little-to-absent inflammatory infiltrate but complement deposition in vessel walls.

IgA, immunoglobulin A; C3, complement component 3.

* Most common form in both COVID-19- and vaccine-associated settings.

** Mostly normocomplementemic.

*** Associated with severe COVID-19 exclusively.

### Cutaneous small-vessel vasculitis

Cutaneous small-vessel vasculitis, also known as leukocytoclastic vasculitis (LCV), is one of the most common CV reported in COVID-19 patients (Supplementary Table 1) (8, 12–24). Nevertheless, it is rare compared to other COVID-19-related dermatological manifestations. Indeed, according to a case-control study on 198 severe COVID-19 patients, LCV accounts for only 1.8% of all cutaneous findings (25). Clinical appearance spans from classic, bilateral symmetric palpable purpura favoring dependent body sites (Supplementary Figures 1a,b) to vesicobullous, hemorrhagic or targetoid eruptions. Oral or intravenous CS, with or without topical CS, were the mainstay of treatment, whereas intravenous immunoglobulin (IVIg) was employed only in a minority of cases (19, 20, 23). As for the prognosis, most patients experienced complete recovery, except for a few who developed vasculitis-associated gangrene or died due to COVID-19-related complications (12, 19, 20, 24).

### IgA vasculitis

IgA vasculitis is a form of small vessel vasculitis characterized by perivascular deposition of hypogalactosylated IgA1 and neutrophil activation, being the most common vasculitis in the pediatric age. Palpable non-thrombocytopenic purpura of lower extremities and buttocks is a characteristic sign of skin-limited IgA vasculitis (IgAV) and Henoch–Schonlein purpura (HSP) (26, 27). Hemorrhagic blisters, as well as targetoid lesions, have been suggested to occur more frequently in skin-limited IgA vasculitis (Supplementary Figure 1c) than cutaneous small vessel vasculitis with IgM/IgG deposits, i.e., LCV (5). Fifteen COVID-19-associated cases (12 males, 3 females), half of which were children, have been reported so far. Palpable purpura (13/15) as well as renal (8/15), gastrointestinal (8/15) and articular (3/15) involvement were documented. In 8 subjects, onset of vasculitis was simultaneous with the infection. All these patients received systemic CS. Biologics and immunosuppressants were administered only in 4 individuals due to concomitant renal impairment (1 rituximab, 2 mycophenolate mofetil, 1 cyclophosphamide), with a favorable response across published reports (28).

### Urticarial vasculitis

Urticarial vasculitis (UV) is a rare clinicopathological entity manifesting with indurated wheal-like lesions lasting more than 24 h (Supplementary Figure 1d) and usually leaving post-inflammatory hyperpigmented *sequelae* upon resolution (29). Although the cause of UV often remains unclear, trigger factors such as drugs, infections, autoimmune diseases, and malignancy have been described (30, 31). Though rare, UV has been described in either symptomatic or asymptomatic COVID-19 patients (32–34). Interestingly, its onset has also been reported a few weeks following recovery from COVID-19 (35). Antihistamines alone or in combination with oral CS were administered in all the subjects (32–35).

### Other vasculitides associated with COVID-19

Anti-neutrophil cytoplasmic antibodies (ANCA) associated vasculitis (AAV) can sometimes mimic COVID-19 in terms of pulmonary involvement and COVID-19 may occur simultaneously with AAV. (36). Six patients, 4 of whom were males, were diagnosed with AAV simultaneously or shortly after COVID-19. Three had serum antibodies directed against myeloperoxidase (anti-MPO), while the others had anti-proteinase 3 (anti-PR3) antibody positivity. Fever, respiratory and gastrointestinal symptoms were reported. All patients survived after adequate treatment with immunosuppressive medications (37).

### COVID-19-associated cutaneous vasculitides in the pediatric age

According to a recent systematic review by Batu et al., which gathered 36 pediatric patients, the median age of onset of vasculitis was 13 years, with a male predominance (M/F: 2.3). The median time from infection to onset of vasculitis was 17.5 days (range: 2–150). Among those with potential skin involvement, the most frequently reported in that pediatric age included IgAV/HSP (25%) chilblains (19.4%), UV (5.5%), cutaneous leukocytoclastic vasculitis (2.7%), and acute hemorrhagic edema of infancy (AHEI, 2.7%) (38).

Kawasaki disease (KD) is an acute systemic vasculitic syndrome primarily affecting children below the age of 5, that involves small and medium-sized vessels with a predilection for coronary arteries (39). As SARS-CoV-2 infection can lead to endothelial inflammation and dysfunction, it can also trigger the development of KD in certain individuals (40). Moreover, older children (median age of 8 years) can be affected by a similarly severe inflammatory disorder with multisystem involvement (MIS-C), also known as Pediatric Multisystem Inflammatory Syndrome temporally associated with SARS-CoV-2 (PIMS-TS) (41). MIS-C is mainly characterized by systemic vasculitis, mucocutaneous inflammatory signs (rash), multisystem involvement, and hypercoagulation, although thrombotic or embolic events were rare, when compared with adult COVID-19 (42). Although it may present a clinical overlap with KD or toxic shock syndrome, it is regarded as a separate entity (43).

### Pathophysiology of COVID-19-associated vasculopathy and vasculitis

Distinct pathomechanisms have been implicated in the genesis of the above-mentioned COVID-19-associated cutaneous findings, depending on the presence/absence of a robust type I interferon signature: (i) transient, true vasculitis in mild cases (e.g., COVID-toes) and (ii) small vessel thromboembolic disease, without true vasculitis (i.e., vasculopathy) in patients with severe disease. A number of other forms may be placed between the two ends of this spectrum (44).

In greater detail, vasculitic changes with lymphocytic perivascular cuffing and infiltration, possibly leading to secondary luminal thrombosis, result from type I interferon responses, akin to familial chilblain lupus or STING-associated vasculopathy with onset in infancy (45, 46).

In contrast, dysfunction of vascular endothelium due to the SARS-CoV-2 infection has been suggested in the pathogenesis of the COVID-19 vasculopathy (47). Initially, it was speculated that endothelial injury was due to direct viral infection (48), but recent evidence demonstrated that endothelial cells present low Angiotensin Converting Enzyme 2 (ACE2) expression and are resistant to SARS-CoV-2 infection, supporting the involvement of an indirect mechanism of endothelial injury in the pathogenesis of COVID-19 vasculopathy (47, 48). This is best exemplified by patient with severe COVID-19 pneumonia. In the lungs, when respiratory and alveolar epithelial cells get infected by SARS-CoV-2 in the setting of defective antiviral interferon signaling, an exacerbated innate inflammatory loop is induced with elevation of Interleukin (IL)-1β, IL-6 and Tumor Necrosis Factor (TNF)α. The subsequent release of several proinflammatory cytokines/chemokines at a systemic level, together with secondary complement activation due to ischemia, leads to indirect endothelial cell injury through loss of their antithrombogenic properties and barrier function (49). This process, known as pulmonary immunothrombosis, possibly followed by pulmonary venous microembolism, may account for the clinico-pathological picture observed in severe COVID-19 cases, with pauci-immune thrombogenic vasculopathy and terminal complement activation in vessel walls (50), but only sporadic SARS-CoV-2 spike protein deposition (44).

Increased levels of galactose deficient IgA1 (gd-IgA1) are necessary for the development of IgA nephritis, with a multi-hit model involving IgA1 and anti-endothelial cell antibodies currently accepted to explain its vasculitic, extrarenal manifestations. Mucosal infections, such as COVID-19, are believed to enhance IL-6 production thereby stimulating poor glycosylation/galactosylation of IgA1 in predisposed subjects. The subsequent formation gd-IgA1 may contribute toward the disease process of IgA vasculitis in a proportion of COVID-19 patients (51).

A humoral response against SARS-CoV-2 antigens, leading to immune-complex formation, could underscore cases of COVID-19-associated urticarial vasculitis and leukocytoclastic vasculitis (52). Indeed, SARS-CoV-2 antigens have been detected in skin biopsies from two UV patients with COVID-19, supporting the existence of a causal link (33). Consistent with a type III hypersensitivity response, circulating immune complexes could act as triggers for classic complement pathway activation, thus promoting neutrophil recruitment, vascular leakage and subsequent vessel wall injury and inflammation (52).

It is noteworthy that anti-phosphatidylserine/prothrombin complex antibodies have been implicated in models of cutaneous vasculitis (53) and that anti-prothrombin antibodies increase after infection with SARS-CoV-2 (54).

Concerning ANCA-associated vasculitides, NETs overproduction has been described during COVID-19. Prolonged exposure of NETs to proteins as well as their reduced clearance may be key in explaining the onset of ANCA autoimmunity in predisposed subjects infected by SARS-CoV-2 (36).

**Figure 1:**
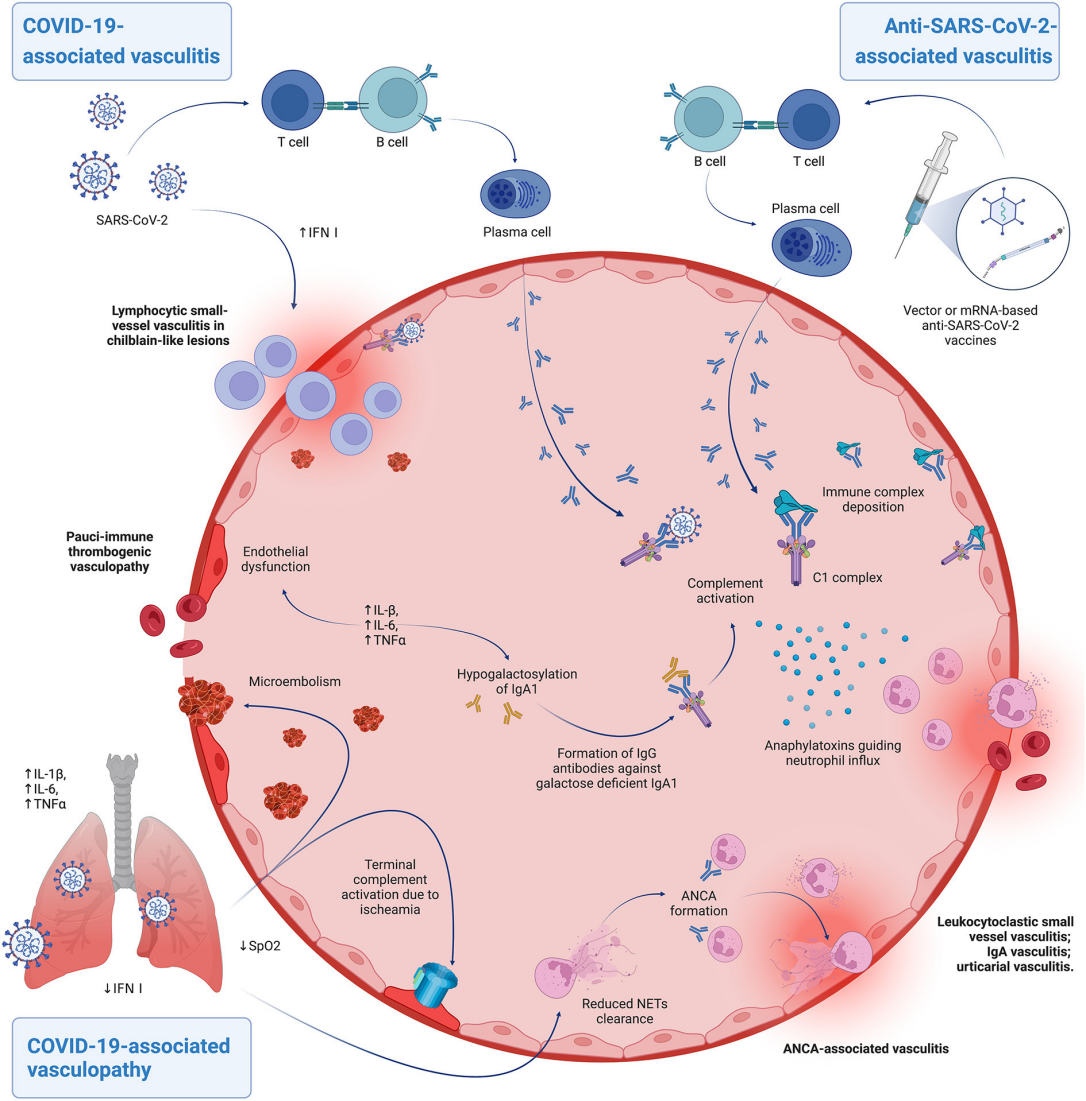
Hypothesized pathomechanisms of COVID-19-associated vasculitis/vasculopathy and anti-SARS-CoV-2-vaccine associated vasculitis. Patients able to mount a robust type I interferon response can develop lymphocytic perivascular cuffing during infection, leading to chilblain-like lesions. The virus can also cause immune complex deposition, with subsequent complement activation and neutrophil recruitment, determining the clinicopathological picture of leukocytoclastic vasculitis. A similar process, possibly with anaphylatoxins inducing exaggerated mastocyte activation, is thought to underscore cases of urticarial vasculitis. Both these forms can occur also in vaccine-related cases. Patients that develop severe COVID-19 due to a defective antiviral response may experience indirect endothelial injury, due to elevated proinflammatory cytokines at a systemic level and ischaemia-induced complement activation. This process is known as pulmonary immunothrombosis and may be complicated by pulmonary venous embolism. Speculatively, the inflammatory *milieu* brought about by the infection can lead to other forms of cutaneous vasculitides. Elevated IL-6 levels may contribute to hypogalactosylation of IgA1 in predisposed individuals triggering an IgG-mediated response that may result in IgA vasculis. Prolonged exposure and reduced clearance of Neutrophil Extracellular Traps (NETs) in the context of COVID-19 dysregulated inflammatory responses may set the basis for ANCA-associated autoimmunity and vasculitis. Created with BioRender.com.

Main proposed pathomechanisms are summarized in Figure 1.

## Anti-SARS-CoV-2 vaccination-induced cutaneous vasculitis

Vasculitides and other vascular affections have also been reported following anti-SARS-CoV-2 vaccination (55).

### Cutaneous small-vessel vasculitis

Skin-limited small vessel vasculitis or LCV (56) represents the most common CV reported after anti-SARS-CoV-2 vaccination. It has been observed after the Pfizer-BioNTech mRNA vaccine (BNT16B2b2) (57–65). Moderna mRNA vaccine (mRNA-1273) (66–68). Oxford-AstraZeneca adenoviral vaccine (ChAdOx1 nCoV-19 AZD1222) (69–79). Johnson & Johnson adenoviral vaccine (Ad26.COV2.S) (80–82), and inactivated vaccines [Sinovac CoronaVac (83), Bharat Biotech Covaxin (84), Sinopharm BBiBP-CorV] (85, 86) (Supplementary Table 2).

Almost every case was biopsy-confirmed. Notably, some of these cases were reported as “immunocomplex vasculitides” (65–68). Some patients experienced systemic symptoms such as joint pain (64, 70, 73, 80, 84), and microhematuria (79, 80). In one patient, gastrointestinal involvement with melena and diarrhea was reported (65). In some cases, cryoglobulins were detected on serological analysis (82, 87); notably, the case by Nastro et al. also featured a concomitant atypical herpes zoster of the right leg (59). Among reviewed cases, one had history of SARS-CoV-2 infection (78) and one had a previous diagnosis of leukocytoclastic vasculitis (58).

Treatment was generally represented by oral CS and antihistamines (local corticosteroids, non-steroid anti-inflammatory drugs (NSAIDs), colchicine, antibiotics, analgesics, pentoxifylline, dapsone were also prescribed in a minority of cases). Spontaneous remission was occasionally reported. All patient recovered in 1–8 weeks, except for one individual who developed COVID-19 20 days after vaccination with Oxford-Astrazeneca ChAdOx1 nCoV-19 vaccine (78). This patient presented with cough, myalgia, and fatigue, and developed progressive skin manifestations, including urticarial and purpuric lesions over her upper and lower extremities and abdomen. After 9 days, she developed multiorgan failure and died. In this case it is likely that the SARS-CoV-2 infection rather than the vaccination could have acted as trigger of the vasculitis (78).

### IgA vasculitis

IgA vasculitis (88) has been observed after vaccination with Pfizer-BioNTech mRNA vaccine (BNT16B2b2) (57, 89–93), Moderna mRNA vaccine (mRNA-1273) (94, 95), Oxford-AstraZeneca adenoviral vaccine (ChAdOx1 nCoV-19 AZD1222) (96–98), and Sinovac inactivated vaccine (CoronaVac) (99) (Supplementary Table 3). Of note, histology was not available in all cases. Some patients also experienced systemic symptoms such as joint (93, 96–98) or abdominal pain (94), hematuria or renal impairment (90, 94, 96). Treatment of choice was generally represented by oral CS, but spontaneous remission was also occasionally reported. All patients recovered in several weeks. Interestingly, of the reviewed cases, two had history of SARS-CoV-2 infection (97, 99); three had history of previous IgA vasculitis (90, 94) or Henoch-Schönlein purpura (92).

### Lymphocytic vasculitis

Lymphocytic vasculitis is a histologic reaction pattern with a dominant lymphocytic inflammatory infiltrate (100). Reported cases of lymphocytic vasculitis followed the inoculation of Pfizer-BioNTech mRNA vaccine (BNT16B2b2) (101), Oxford-AstraZeneca adenoviral vaccine (ChAdOx1 nCoV-19 AZD1222) (102), inactivated vaccine Bharat Biotech Covaxin (103) and mRNA-1273 Moderna vaccine (104) (Supplementary Table 4).

Treatment of choice was generally represented by oral antihistamines or local CS (one case was managed with follow up only). All patients fully recovered in 2 weeks. Notably, one of the patients had SARS-CoV-2 infection before vaccination (101).

### Urticarial vasculitis

Cases of UV were reported after Moderna mRNA vaccine (mRNA-1273) (99, 105), Oxford-AstraZeneca adenoviral vaccine (ChAdOx1 nCoV-19 AZD1222) (106), and inactivated vaccine Sinovac CoronaVac (107) (Supplementary Table 5). Treatment of choice was generally represented by oral corticosteroids, but oral antihistamines, dapsone and indomethacin were also used. All patient fully recovered in 1–8 weeks, this being in line with the expected course of drug-induced UV (31).

### Other vasculitides: ANCA-associated vasculitis and other forms

Only one case of AAV presenting with cutaneous involvement has been reported following anti-SARS-CoV-2 vaccination (Pfizer-BioNTech mRNA vaccine) (BNT16B2b2) (108). Interestingly, the patient had been taking propylthiouracil for Graves' disease and therefore her condition was identified as a propylthiouracil-induced ANCA-associated vasculitis. In this case, the treatment of choice was represented by oral corticosteroids and the patient recovered after 3 weeks. Of note, there are case reports of AAV with systemic involvement and absence of cutaneous features, triggered by anti-SARS-CoV2 vaccination (80, 109).

Among the unclassifiable forms, we also describe the peculiar vasculitis reported by Nasr et al. The 64-year-old female patient had history of Raynaud's disease, hand arthritis, photosensitivity, Sjogren's syndrome and leukocytoclastic vasculitis; 3 days after receiving the first dose of Pfizer–BioNTech mRNA vaccine she developed fingertip necrosis (caused by a type II cryoglobulinemia) and a new episode of purpuric rash on the lower extremities (likely leukocytoclastic vasculitis). The workup revealed cryoglobulinemia, hypocomplementemia, elevated antinuclear antibodies and IgM antiphospholipid autoantibodies, suggesting a diagnosis of systemic lupus erythematosus and antiphospholipid syndrome (110).

Lastly, multisystem inflammatory syndrome (MIS) after vaccination deserves a brief mention. MIS has been associated with SARS-CoV-2 infection, has a latency of 4–6 weeks, and can be ultimately described as a vasculopathy clinically resembling Kawasaki disease and potentially leading to acute cardiac dysfunction and multiorgan failure (111, 112). It usually occurs in children (MIS-C), but adult forms are also reported (MIS-A) (112). Interestingly, very rare cases of MIS are reported in absence of viral infection, after anti-SARS-CoV-2 vaccination (MIS-V) (113). In children, MIS-V had a frequency of 1.5 cases per million of injected doses, with patients aged 12–20 years, presenting with fever, coagulopathy, mucocutaneous, cardiac, gastrointestinal, and renal involvement. They were treated with systemic CS and/or IGIV and had a favorable outcome (111, 114). Similarly, adult MIS-V forms are also reported, with comparable course, treatment, and outcome (112, 115–118) (Supplementary Table 6).

### Pathophysiology of anti-SARS-CoV-2-vaccine-associated vasculopathy and vasculitis

Immune-complex deposition with ensuing complement activation is currently regarded as the plausible pathophysiology for most anti-SARS-CoV-2-vaccination-associated cutaneous vasculitides (119). SARS-CoV-2 vaccine components sharing structural similarities to host proteins may promote a pro-inflammatory state followed by the activation of autoreactive B/T cells, antibody formation, and subsequent immune complex deposition in the small vessels of the skin with potential involvement of internal organs as well (120). Molecular mimicry phenomena may also have a role. An unrelated antigen or an underlying genetic predisposition unmasked, due to the vaccine's immune enhancing properties, should be considered as well.

## Conclusions

In conclusion, we reviewed available evidence on COVID-19-associated and anti-SARS-CoV-2-vaccine-associated CV, showing superimposable findings with pre-pandemic cases.

Although IgA immune deposits were prevalent, lack of reporting of immunofluorescence findings in most papers hinders a thorough analysis of above-mentioned cases and calls for further studies, as the very nature of immune deposits in the non-COVID-19/COVID-19-vaccine-associated setting is still debated (5).

Patients referred for purpuric lesions often pose a challenge to dermatologists and many algorithms have been proposed to simplify their differential diagnosis and thereby stratify their prognosis (121). Signs of retiform (i.e., branched) purpura at acral sites or generalized, particularly, have been suggested to portend poor prognosis in patients with complex purpura (121) and, though they may be lacking validation in this specific scenario, they could pose as a useful clue also in COVID-19 patients, to promptly recognize those at a higher risk of immunothrombotic vasculopathy.

Adequately assessing the causal link on an individual case basis along with thorough patient counseling should aim to minimize vaccine hesitancy, as seen in other vaccine-associated dermatological conditions (122).

Despite the wealth of clinical evidence available concerning COVID-19-associated and anti-SARS-CoV-2-vaccine-associated cutaneous vasculitis and vasculopathy, there is a paucity of studies addressing the pathophysiology of these manifestations. Further research is therefore needed to inform pathogenesis-driven treatment.

## Author contributions

EZ, GA, CM, MR, and CAM reviewed the pertaining literature and wrote the manuscript. AM, SR, and PQ supervised the draft. CAM and AM edited and approved the final draft. All authors have made substantial contribution to the work and have approved the final version of this article.

## Funding

Publication costs were partially funded by Grant Ricerca Corrente / Finalizzata, Italian Ministry of Health.

## Conflict of interest

The authors declare that the research was conducted in the absence of any commercial or financial relationships that could be construed as a potential conflict of interest.

## Publisher's note

All claims expressed in this article are solely those of the authors and do not necessarily represent those of their affiliated organizations, or those of the publisher, the editors and the reviewers. Any product that may be evaluated in this article, or claim that may be made by its manufacturer, is not guaranteed or endorsed by the publisher.
