# Supplementary figures and images for: Cutaneous vasculitis and vasculopathy in the era of COVID-19 pandemic
Source: Front Med (Lausanne). 2022 Aug 23;9:996288. doi: 10.3389/fmed.2022.996288 (PMC9445267; doi:10.3389/fmed.2022.996288)

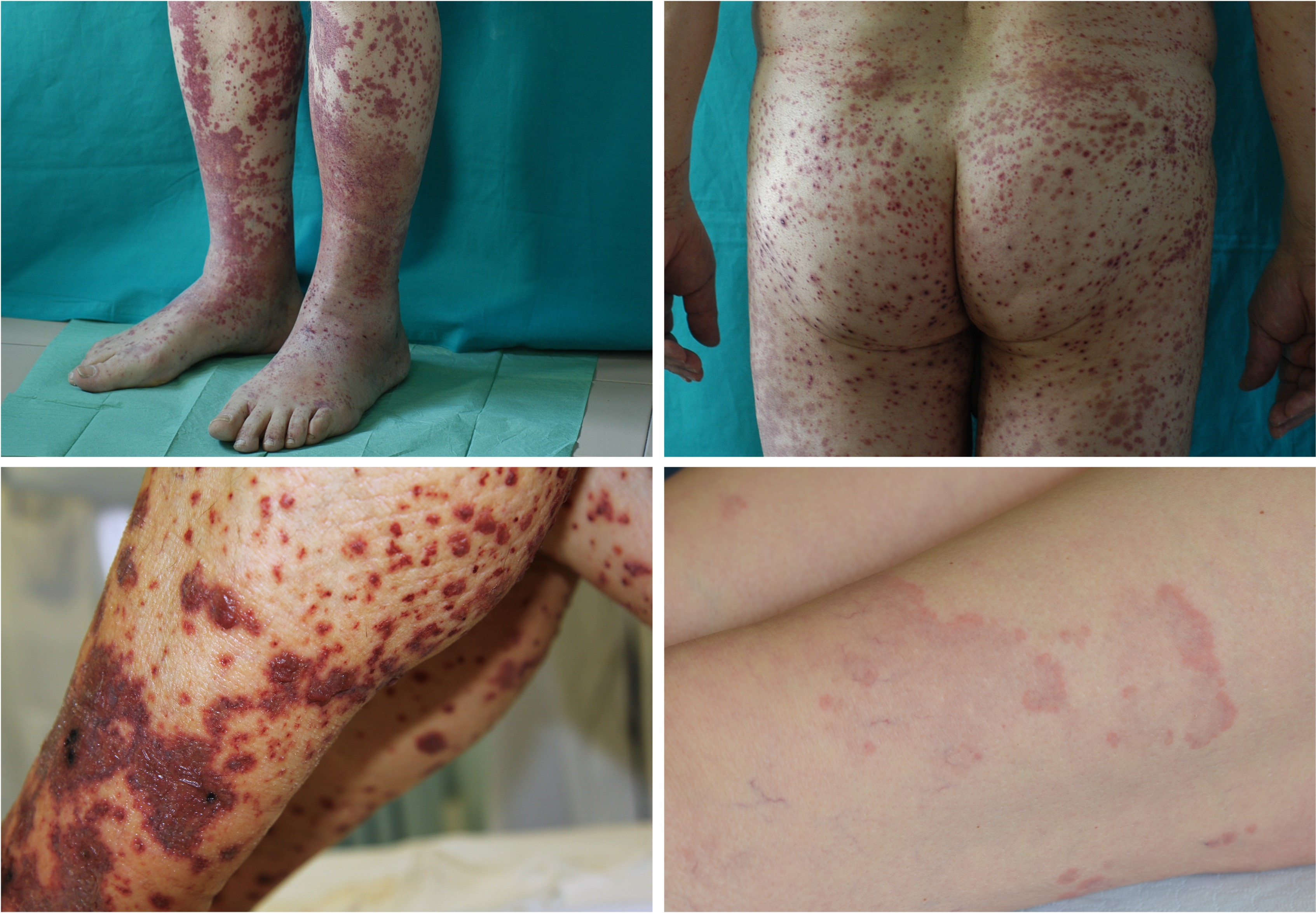

Supplement: Supplementary file 1 [file Image_1.JPEG]
