# Supplementary material for: Cutaneous vasculitis and vasculopathy in the era of COVID-19 pandemic
Source: Front Med (Lausanne). 2022 Aug 23;9:996288. doi: 10.3389/fmed.2022.996288 (PMC9445267; doi:10.3389/fmed.2022.996288)
Supplement: Supplementary file 2 [file Data_Sheet_1.pdf]

Supplementary Materials

Supplementary Figure legends.

Supplementary Figure 1. Classic, palpable non-blanchable purpura involving the legs (a, upper left) and buttocks (b, upper right) in a patient with small-vessel cutaneous vasculitis. Haemorrhagic blisters (c, lower left) in a patient with skin-limited IgA vasculitis. Infiltrated, polycyclic wheal-like lesions lasting more the 24 hours in a patient with urticarial vasculitis (d, lower right).

Supplementary Table 1. Cases of histologically confirmed cutaneous leukocytoclastic vasculitis in COVID-19 patients

Ref.	Gender, age (years)	Clinical findings	Systemic involvement	Autoantibodies	COVID-19 diagnosis (methods)	Treatment	Time to resolution	Outcome
(12)	M, 79	Haemorrhagic vesiculobullous eruption on neck and dorsal areas of hands	Fever and dyspnea	Negative	Nasopharyngeal swab RT- PCR	Intravenous CS	NA	Death for respiratory insufficiency
(13)	M, 59	Symmetrically distributed maculopapular purpuric exanthema on the face, trunk and extremities	Neurological complications	Negative	Throat swab RT-PCR	NA	NA	NA
(14)	M, 29	Purple palpable papules and serohaematic blisters on the abdomen, buttocks and both lower legs	Myalgia, abdominal pain, diarrhea	ANA 1/40	Positive SARS-CoV-2 PCR from skin biopsy	CS	15 days	Recovery
(15)	M, 47	Small red to violaceous plaques on the legs and extended to the arms and trunk	Fever arthralgia, oral lesions	Negative	Positive RT-PCR	Topical betamethasone valerate 0.12% cream twice daily	NA	NA
(16)	F, 71	Purpuric macules and papules in both legs, extending from the ankle and up to the thigh	No	Negative	Positive rRT-PCR for SARS-CoV-2 from nasopharyngeal swab	Betamethasone dipropionate 0.05% cream	3 weeks	Recovery
(17)	F, 83	Purple palpable papules and serohaematic blisters on lower legs, feet and toes	No	IgM cardiolipin antibodies slightly increased	Serological qualitative rapid testing for SARS- CoV-2 positive for IgM and IgG antibodies	30 mg/day prednisone	NA	Recovery
(18)	M, 49	Purpuric lesions on flexor and extensor parts of both legs and thighs and lower abdomen	No	Negative	Positive SARS-Cov 2 test *	NA	NA	NA
(19)	M, 41	Purpuric lesions on legs and livedo reticularis	Cardiac,renal, coagulation profile	Positive Lupus anti-coagulant	Positive SARS-CoV-2 test from nasopharyngeal swab*	IGIV	NA	Dry gangrene and amputation of the left big toe
(20)	M,13	Rashes over the lower limbs	No	Mildly elevated IgA	Positive RT-PCR test from nasopharyngeal swab 1 month prior to presentation	Oral prednisolone (1 mg/kg per day) for 2 weeks followed by gradual tapering over the next 4 weeks.	4 weeks	Recovery
(21)	M,64	Purpuric macules, papules, and bullae involving his lower extremities with initial aspects of central ulceration and necrosis	No	Negative	positive SARS-Cov 2 test *	Oral prednisone, high-dose IVIG	NA	Death two weeks later due to heath failure
(22)	M,27	Tense vesiculobullous and purpuric lesions over bilateral lower extremities	No	Negative	Positive rRT-PCR for SARS-CoV-2 from nasopharyngeal swab	Prednisone 1mg/kg/day	NA	Recovery
(23)	M, 66	Purplish palpable purpuric papules, some of which with a necrotic center and erythematous maculo- papular lesions on the legs and forearms bilaterally	Abdominal pain, arthralgia, and fatigue	Negative	Positive RT-PCR test from nasopharyngeal swab	Systemic CS (1 mg/ kg per day) and antihistamines.	Complete resolution occurred within 2 weeks.	Recovery
(24)	F, 32	Multiple erythematous to violaceous macules and papules over her bilateral lower extremities involving the dorsum of her feet	No	Antinuclear antibody was detected at high titers of 1:2560	Nasopharyngeal swab RT- PCR	IVIG	NA	Recovery
(25)	M,93	Purpuric macules and papules on the legs, hands, and periumbilical area	Legs, feet, and hands were swollen	Negative	Nasopharyngeal swab RT- PCR	Intravenous methylprednisolone (125 mg daily for three days), clobetasol propionate cream	NA	Dry gangrene of his legs and feet, amputation was declined. The patient died seven weeks after the initial diagnosis of COVID-19 infection.

M= male; F= female; NA= not available**;** CS=corticosteroids; IVIG= Intravenous immunoglobulin; rRT-PCR= Real-time reverse transcription polymerase chain reaction; RT-PCR= Real-time transcription polymerase chain reaction; IVIG= intravenous immunoglobulin *method not specified

Supplementary Table 2. Cases of cutaneous leukocytoclastic vasculitis caused or reactivated by anti-SARS-CoV-2 vaccination

Ref.	Gender, age (years)	Clinical findings	Time to onset (days)	Time to resolution (weeks)	Which dose	Outcome	Treatment	Vaccine type
(58)	F,46	Erythematous palpable papules on the legs (previous leukocytoclastic vasculitis)	2	Several	First and second	Recovery	Prednisolone 20 mg/day for 2 weeks	Pfizer-BioNTech BNT16B2b2
(59)	M,48	Palpable purpura on both legs	4	3	Second	Recovery	Systemic antihistamines and local CS	Pfizer-BioNTech BNT16B2b2
(60)	M,84	Palpable purpura on the legs, contemporary herpes zoster of the leg	1	2	First	Recovery	Famciclovir 500 mg 3/day for 10 days and follow up	Pfizer-BioNTech BNT16B2b2
(61)	F,57	Widespread erythematous eruption involving the trunk and extremities	7	1	First	Recovery	Prednisolone 20 mg/day for 1 week	Pfizer-BioNTech BNT16B2b2
(62)	M,65	Palpable purpuric lesions on the legs	2	Several	Third	Recovery	Prednisolone 60 mg/day for 1 week, topical steroid and mupirocin	Pfizer-BioNTech BNT16B2b2
(63)	F,42	Progressive rash on the lower legs and the gluteal region	4	1	First	Recovery	Prednisolone 60 mg/day	Pfizer-BioNTech BNT16B2b2
(64)	F,22	Purpuric rash on the legs (no biopsy)	7	3	Second	Recovery	Prednisolone 25 mg/day for 1 week, oral antihistamines	Pfizer-BioNTech BNT16B2b2
(65)	M,83	Palpable purpura on legs	4	2	First	Recovery	Methylprednisolone, systemic antihistamines,	Pfizer-BioNTech BNT16B2b2
(66)	M,76	Purpuric rash with palpable purpura on both hands, legs, thighs, and abdomen (no biopsy, diagnosed as immune complex vasculitis)	12	3	Second	Recovery	Prednisolone 40 mg/day for 2 weeks	Pfizer-BioNTech BNT16B2b2
(67)	F,59	Purpuric rash involving the lower extremities and abdomen (no biopsy)	1	NA	Second (previous SARS-CoV-2 infection)	Recovery	Oral antihistamines and CS	Moderna mRNA-1273
(68)	M,52	Ppurpuric rash on the root of the lower limbs	11	NA	Second	Recovery	No treatment	Moderna mRNA-1273
(69)	20s	Skin purpura (diagnosed as immune complex vasculitis)	8	/	/	Recovery	Local CS	Moderna mRNA-1273
	60s		9	/	/		Systemic CS	Oxford-AstraZeneca ChaAdOx1 nCoV-19
	40s		8	/	/		NSAID	Oxford-AstraZeneca ChaAdOx1 nCoV-19
(70)	F,60	Painful purpuric lesions and palpable papules on the lower limbs	11	2	Second	Recovery	Prednisone 60 mg/day for 2 weeks	Oxford-AstraZeneca ChaAdOx1 nCoV-19
(71)	F,55	Palpable purpura over both ankles, that progressed to the lower limb	5	2	First	Recovery	Prednisolone 0.5 mg/kg/day, for 2 weeks	Oxford-AstraZeneca ChaAdOx1 nCoV-19
	M,48		2		First and second		Topical CS	
(72)	F,68	Erythematous macules on both lower extremities	2	3	First	Recovery	Oral methylprednisolone, colchicine 1.2 mg/day and topical CS for 3 weeks	Oxford-AstraZeneca ChaAdOx1 nCoV-19
(73)	M,57	Erythematous macules on both lower extremities and torso	14	3	First	Recovery	Prednisone 1 mg/kg/day for 3 weeks	Oxford-AstraZeneca ChaAdOx1 nCoV-19
	M,58		7	10 days	Second			
	M,53		6	2	First			
(74)	F,62	Petechial rash on the bilateral lower limb	7	Several	First	Recovery	Oral prednisone	Oxford-AstraZeneca ChaAdOx1 nCoV-19
(75)	F,57	Palpable purpura lesions on the buttocks	1	1	First	Recovery	Only follow-up	Oxford-AstraZeneca ChaAdOx1 nCoV-19
(76)	F,77	Palpable papules on the lower limb and the hands	10	Several	First	Recovery	Oral prednisolone, dapsone 50 mg/day for 60 days	Oxford-AstraZeneca ChaAdOx1 nCoV-19
(77)	F,71	Palpable purpuric papules on the legs	5	2	Second	Recovery	Oral prednisone 20 mg/day.	Oxford-AstraZeneca ChaAdOx1 nCoV-19
(78)	F,61	Pruritic erythematous-purpuric macules involving the lower extremities, buttocks, axillae, and lateral aspects of the abdomen	5	2	First	Recovery	Oral antihistamines and CS	Oxford-AstraZeneca ChaAdOx1 nCoV-19
(79)	F,56	Purpuric rash involving the lower extremities and abdomen	30	9 days	First and SARS-CoV-2	Death	Oral CS, norepinephrine, antibiotic and antiviral therapy	Oxford-AstraZeneca ChaAdOx1 nCoV-19

					infection 20 days later			
(80)	F,64	Palpable purpuric papules on the lower limbs	1	2	First	Recovery	Oral antihistamines and topical CS	Oxford-AstraZeneca ChaAdOx1 nCoV-19
	F,44		2	3	Second	Recovery	Oral antihistamines and CS, topical CS	
	F,68		9	10 days	First	Recovery	Oral antihistamines and CS, topical CS	
	F,67		20	2	First	Recovery	Oral antihistamines, CS, and pentoxifylline, topical CS	
	F,59	Palpable purpuric papules on the lower and upper limbs	2	4 days	First	Partial resolution	Oral antihistamines and CS, topical CS	
(81)	M,22	Palpable purpura on the dorsal feet and the both lower extremities	10	3	First	Recovery	Oral antihistamines and local CS	Johnson & Johnson Ad26.COV2.S
(82)	M,65	Pruritic and painful purpuric rash on his left arm, lower extremities, and abdomen	7	8	First	Recovery	Prednisone 60 mg/day and topical CS, analgesics	Johnson & Johnson Ad26.COV2.S
(83)	F,30	Purpuric and hemorrhagic papules and vesicles on feet, legs and elbows	17	Several	First	Recovery	Topical CS	Johnson & Johnson Ad26.COV2.S
(84)	F,23	Erythematous pinpoint purpura on the lower and upper extremities	36	4	First	Recovery	Dexametasone and prednisolone	Sinovac CoronaVac
	F,26		4 hours				Oral colchicine 0.6 mg twice a day and naproxen	
(85)	F,46	Purpuric papules on the legs	5	2	First	Recovery	Systemic antihistamines and local steroid	Bharat Biotech Covaxin
(86)	M,45	Papular lesions on upper and lower limbs	2	NA	First	Recovery	Systemic CS	Sinopharm BBiBP-CorV
(87)	F,57	Palpable purpuric eruption on legs, thighs, buttocks, abdomen, back, and both forearms	5	6	Second	Recovery	Systemic CS	Sinopharm BBiBP-CorV
(88)	F,83	Palpable purpura on both hands, legs and thighs	7	2	Second	Recovery	Oral antibiotic and topical CS	Pfizer-BioNTech BNT16B2b2

M= male; F= female; NA=not available; CS=corticosteroids.

Supplementary Table 3. Cases of cutaneous IgA vasculitis caused or reactivated by anti-SARS-CoV-2 vaccination

Ref.	Gender, age (years)	Clinical findings	Time to reaction (days)	Time to resolution (weeks)	Which dose	Outcome	Treatment	Vaccine type
(58)	F,17	Palpable purpura on both arms and legs	10	4	First dose	Recovery	Systemic antihistamines and local steroid	Pfizer-BioNTech BNT16B2b2
(90)	F,40	Gluteal region rash (no biopsy)	20	1	Second dose	Recovery	Only follow-up	Pfizer-BioNTech BNT16B2b2
(91)	M,23	Palpable purpuric rash on the extremities and trunk (no biopsy, previous IgA vasculitis)	1	2	Second dose	Recovery	Prednisone 20 mg/day for 2 weeks	Pfizer-BioNTech BNT16B2b2
(92)	F,70	Palpable purpura on the feet	2	3	Second dose	Recovery	Only follow-up	Pfizer-BioNTech BNT16B2b2
(93)	F,45	Palpable purpura on the feet (no biopsy, previous Henoch-Schonlein purpura)	1	4	Second dose (reported also 6 days after the third dose)	Recovery	Only follow-up	Pfizer-BioNTech BNT16B2b2
(94)	M,34	Widespread palpable purpura	7	2	Second dose	Recovery	Prednisolone 40 mg/day	Pfizer-BioNTech BNT16B2b2
(95)	F,78	Palpable purpura on the hips and lower limbs (no biopsy, previous IgA vasculitis with leukocytoclastic aspects, and renal and gastrointestinal involvement)	7	2	First dose	Recovery	Methylprednisolone 1 mg/kg/day for 2 weeks	Moderna mRNA-1273
(96)	M,94	Palpable purpura on the waist	10	Several	Second dose	Recovery	Prednisone 60 mg/day for 3 weeks	Moderna mRNA-1273
(97)	F,76	Maculopapular purpuric rash on gluteal and leg regions	7	3	First dose	Recovery	Deflazacort 30 mg/day for 10 days	Oxford-AstraZeneca ChaAdOx1 nCoV-19
(98)	M,62	Petechial purpuric rashinvolving both legs	8	1	First dose	Recovery	Prednisone 40 mg/day for 7 days	Oxford-AstraZeneca ChaAdOx1 nCoV-19
(99)	M,72	Maculopapular rashlower limbs	15	Several	First dose	Recovery	Prednisone 40 mg/day for 3 weeks	Oxford-AstraZeneca ChaAdOx1 nCoV-19
(100)	M,33	Erythematous macules and palpable papules on the legs, forearms	3	2	First dose (previous infection 3 months before)	Partial resolution	Topical CS cream	Sinovac CoronaVac

M= male; F= female; NA= not available; CS=corticosteroids.

Supplementary Table 4. Cases of cutaneous lymphocytic vasculitis caused by anti-SARS-CoV-2 vaccination

Ref.	Gender, age (years)	Clinical findings	Time to reaction	Time to resolution	Which dose	Outcome	Treatment	Vaccine type
(102)	F,51	Maculopapular rash on upper limbs and trunk	1 day	2 weeks	First (previous SARS-CoV-2 infection)	Recovery	Systemic antihistamine and local CS	Pfizer-BioNTech BNT16B2b2
(103)	M,64	Maculopapular rash on the limbs and trunk	3 days	2 weeks	Second	Recovery	Only follow-up	Oxford-AstraZeneca ChaAdOx1 nCoV-19
(104)	F,31	Purpuric lesions on her legs	4 days	1 week	First and second	Recovery	Systemic antihistamines	Bharat Biotech Covaxin
(105)	M,16	maculo-papul ar rash on the lower limbs and forearms	3 days	2 weeks	Third	Recovery	Only follow-up	Pfizer-BioNTech BNT16B2b2 first and second dose, mR NA-1273 Moderna third dose

M= male; F= female; NA= not available

Supplementary Table 5. Cases of urticarial vasculitis caused by anti-SARS-CoV-2 vaccination

Ref.	Gender, age (years)	Clinical findings	Time to reaction (days)	Time to resolution (weeks)	Which dose	Outcome	Treatment	Vaccine type
(106)	F,27	Maculopapular rash on both hands, legs and trunk	10	8	First	Recovery	Systemic methylprednisolone	Moderna mRNA-1273
(100)	F,35	Palpable purpura on both hands, legs and thighs	1	Several	First	Recovery	Systemic antihistamines, methylprednisolone, and dapsone	Moderna mRNA-1273
(107)	M,78	Urticarial plaques over the extremities	7	4	First	Recovery	Oral methylprednisolone	Oxford-AstraZeneca ChaAdOx1 nCoV-19
(108)	M,27	Urticarial plaques over the trunk and extremities	7	1	Second	Recovery	Oral indomethacin 75 mg/day and oral antihistamines	Sinovac CoronaVac

M= male; F= female; NA= not available

Supplementary Table 6. Cases of ANCA-associated vasculitis and other unclassifiable forms caused by anti-SARS-CoV-2 vaccination

Ref.	Gender, age (years)	Clinical findings	Type of vasculitis	Time to reaction (days)	Time to resolution (weeks)	Which dose	Outcome	Treatment	Vaccine type
(109)	F,37	Erythema on forearms, thighs, forehead one ear	ANCA-associated vasculitis	12	3	First	Partial resolution	Prednisolone 30 mg/day for 3 weeks	Pfizer-BioNTech BNT16B2b2
(111)	F,64	Purpuric rash on the legs and necrosis of fingertips (previous history of Raynaud's phenomenon, Sjogren's syndrome and leukocytoclastic vasculitis)	Leukocytoclastic vasculitis and type II cryoglobulinemia vasculitis in the context of systemic lupus erythematosus (SLE) and antiphospholipid syndrome (APS).	17	10	First	Recovery	Oral steroid, heparin, epoprostenol, plasma exchange.	Pfizer-BioNTech BNT16B2b2

M= male; F= female; NA= not available
